# Molecular Diagnostics and Pathogenesis of Fungal Pathogens on Bast Fiber Crops

**DOI:** 10.3390/pathogens9030223

**Published:** 2020-03-18

**Authors:** Yi Cheng, Xiaoyu Tang, Chunsheng Gao, Zhimin Li, Jia Chen, Litao Guo, Tuhong Wang, Jianping Xu

**Affiliations:** 1 Institute of Bast Fiber Crops and Center of Southern Economic Crops, Chinese Academy of Agricultural Sciences, Changsha 410205, China; chengyi@caas.cn (Y.C.); qifp312@csu.edu.cn (X.T.); gaochunsheng@caas.cn (C.G.); lizhimin@caas.cn (Z.L.); chenjia01@caas.cn (J.C.); guolitao@caas.cn (L.G.); wangtuhong@caas.cn (T.W.); 2 Department of Biology, McMaster University, Hamilton, L8S 4K1, Canada

**Keywords:** bast fiber crops, molecular identification, fungal disease, DNA barcode, PCR assay

## Abstract

Bast fibers and products derived from them are undergoing a resurgence in demand in the global market. However, fungal diseases have become an important factor limiting their yield and quality, causing devastating consequences for the production of bast fiber crops in many parts of the world. Thus, there is a high demand for effective control and prevention strategies against fungal pathogens. Having rapid, specific, sensitive, and cost-effective tests that can be used for early and accurate diagnosis of disease agents is an essential step of such strategies. The objective of this study was to review the current status of research on molecular diagnosis of fungal pathogens on bast fiber crops. Our search of PubMed identified nearly 20 genera of fungal pathogens on bast fiber crops, among which the five most common genera were *Colletotrichum*, *Pythium*, *Verticillium*, *Fusarium*, and *Golovinomyces*. The gene regions that have been used for molecular identifications of these fungi include internal transcribed spacer (ITS), translation elongation factor 1-α (*EF-1α*), ß-tubulin, calmodulin (*CAL*), histone subunit 3 (*H3*), glyceraldehydes-3-phosphate dehydrogenase (*GAPDH*), etc. We summarize the molecular assays that have been used to identify these fungi and discuss potential areas of future development for fast, specific, and accurate diagnosis of fungal pathogens on bast fiber crops.

## 1. Introduction

Plant infectious diseases are among the most important constraints on the quality and yield of crops. It is estimated that plant diseases cause losses of 10%–15% of the world’s major crops, with direct economic losses of up to hundreds of billions of dollars each year. About 70%–80% of crop diseases are caused by fungal pathogens and the damage can be very serious, significantly reducing the yield and quality of many staple food crops and economic crops like fruits, vegetables, and fiber crops [1]. In addition, several fungal pathogens can secrete a variety of toxins and metabolites harmful to humans and animals, posing a great threat to the safety of agricultural products [2]. At present, most control measures against plant fungal pathogens rely on the applications of broad-spectrum fungicides. However, such fungicides not only increase production costs, but also can bring problems such as environmental pollution, fungicide resistance, and persistent residues on foods and other consumer goods with further implications for human health. In order to minimize the damage to crops caused by fungal diseases, as well as to maximize productivity and ensure agricultural sustainability, early detection and quantification of fungal pathogens is essential for disease prevention and control. However, conventional protocols based on morphological and physiological methods are time-consuming, require significant experience, and may not be sensitive and specific for individual pathogens [3]. Moreover, many fungal pathogens can remain latent in “sub-infection” stages with no obvious symptoms and/or in low numbers, making them difficult to detect, and causing confusion with their roles in diseases. These issues can contribute to delayed or wrong control measures.

During the last three decades, to overcome these problems and minimize crop losses caused by fungal diseases, a diversity of DNA molecule-based tools has been developed for the detection and identification of fungal pathogens. These techniques include conventional polymerase chain reaction (PCR) [4], quantitative PCR (qPCR) [5,6], immunocapture-PCR (IC-PCR) [7,8], droplet digital PCR (dd-PCR) [9], loop-mediated isothermal amplification (LAMP) [10], multiplex tandem PCR [11], fluorescence in situ hybridization (FISH) [12], and DNA microarrays [3]. These methods are typically faster and more accurate than those based on colony morphology, microscopic features, and/or physiological/biochemical characters of pure fungal cultures. Indeed, methods targeting DNA sequences have been applied to detect pathogens during crops’ growth, harvest and postharvest processing stages [13]. Moreover, they have also enabled a deeper understanding of microbial populations and communities associated with crops, especially the microorganisms that are difficult or impossible to cultivate in the lab. Together, technological advances and developments in DNA molecule-based methods have allowed fast and accurate detection and quantification of several fungal pathogens simultaneously in many important crops [14,15]. Information resulting from such work has been used to improve disease control and prevention with more rational decisions about the choice of fungicides to use, the appropriate cultivar(s) to plant, and necessary sanitary measures to apply during various stages of the crop production and processing cycle [16,17,18,19].

The objectives of this review were to identify fungal pathogens associated with bast fiber crops and reveal the molecular methods that ensured their identifications. To accomplish this objective, we searched the PubMed database for papers in this field using key words such as “fungal pathogen”, “bast fiber crop”, and “molecular diagnosis”. In addition, references cited in the initially retrieved articles were further screened for their relevance to our review. All papers retrieved in our searches that used molecular methods to analyze fungal pathogens isolated from bast fiber crops were included in this review. In the sections below, we first describe bast fiber crops (Section 2). This is then followed by descriptions of fungal pathogens identified so far from bast fiber crops (Section 3). In the fourth section, we describe the development and evolution of molecular identification of bast fiber fungal pathogens, with a focus on the timeline and markers used to study fungal pathogens. In the fifth section, we focus on the specific target DNA and the molecular assays that have been used to identify different groups of fungal pathogens on bast fiber crops. We finish by providing a brief summary of the progress so far and discuss potential future areas of research and development.

## 2. Bast Fiber Crops

Bast fiber crops are an important group of economic crops for the purpose of harvesting fibers from stems [20]. These fibers are sclerenchyma fibers associated with the phloem of plants. They arise either with primary tissues from the apical meristem, or with secondary tissues produced by the lateral meristem. Bast fiber is one of four major types of natural plant fibers, with the other three being leaf fiber (e.g., banana and pineapple fibers), fruit and seed fiber (e.g., cotton and coconut fiber), and stalk fiber (e.g., straw fiber from rice, wheat, and bamboo). Bast fiber crops comprise six main species (flax, hemp, ramie, kenaf, jute, and sunn hemp) that are broadly cultivated (Table 1) as well as a few others (kudzu, linden, milkweed, nettle, okra, and paper mulberry) with more limited fiber production [21]. Table 1 summarizes the main bast fiber crops, including their geographic distributions, habitats, commercial use, and main fungal diseases. 

Most bast fiber crops have good fiber strength and are often used to make ropes, twine, packaging materials, and industrial thick cloth [22]. Although the commercial importance of bast fibers has been challenged by the rapid growth of other natural fibers (including animal fibers) and chemical fibers from petroleum, there have been renewed interests in bast fibers in recent years. The renewed interests are driven by several factors, including the bast fibers being a renewable resource for producing high-strength and lightweight composite materials for the textile, construction, and automobile industries. In addition, high contents of crude protein, unsaturated fatty acid, and functional compounds that are beneficial for human health have been found in the seeds, flowers, and leaves of these crops. As a result, these crops have been the source of materials for making a diversity of functional feeds for animals, as well as foods, food additives, and therapeutic drugs for humans [22]. An example of functional compounds is cannabinoids in industrial hemp that are attracting broad attention from both healthcare professionals and the general public.

The increasing medical interests and commercial demands for bast fiber crops have resulted in expanding areas for growing bast fiber crops and changing cultivation practices. For example, it is now common to use the same piece of land continuously to grow the same bast fiber crop. However, in such a situation, certain disease agents will likely be enriched in the crop fields, causing increasingly severe diseases to the crops. In the last twenty years, dozens of fungal diseases in bast fiber crops have been identified. The loss of productivity due to fungal diseases was estimated at 10%–50%, with variations attributed to crop type, geographic region, size of planting area, and growth cycle of the crop (especially for continuously growing fields) [22]. In addition, fungal infections also damage the quality of bast fiber and reduce their commercial value. Therefore, having a stable and predictive crop production is crucial for the healthy development of the whole industry. Furthermore, having an early and accurate diagnosis of fungal pathogens infecting bast fiber crops would contribute to disease surveillance and to the implementation of a rational disease management strategy for these crops. Based on the findings retrieved from PubMed, below we review the main fungal disease agents of bast fiber crops, and the principal molecular markers and assays that have been used for detecting fungal pathogens of these crops.

## 3. Fungal Pathogens of Bast Fiber Crops

As shown in Table 1, most bast fiber crops can grow in a diversity of geographic regions and ecological niches. However, some of them have relatively limited geographic and/or ecological distributions and can’t grow well in certain environments. As a result, the types of land used to cultivate certain bast fiber crops may be limited and the same fields may be used to grow the same crop over many years. Even for bast fiber crops with broad ecological adaptability, the limited agricultural land in certain regions and the drive to seek high commercial benefits often mean that only certain types of fields are used for growing each specific crop. In these fields, fungal infectious diseases often increase over time, leading to large yield loss, or even total destruction of the harvest. Fungal pathogens occurring on bast fiber crops are taxonomically very broad (Table 2). Below we describe the major genera and species of fungal pathogens impacting bast fiber crops.

Fungi from the ascomycetous genus *Colletotrichum* cause anthracnose disease in a wide range of plant species, often resulting in significant economic losses [23]. The following six *Colletotrichum* species have been reported from bast fiber crops: *Colletotrichum phormii*, *Colletotrichum fructicola*, *Colletotrichum siamense*, *Colletotrichum corchorumcapsularis*, *Colletotrichum higginsianum*, and *Colletotrichum gloeosporioides*. These species have been reported from flax, jute, kenaf, and ramie, causing an average crop loss of about 20%, with certain crop losses up to 50%. The anthracnose diseases on bast fiber crops have been reported from the US, Australia, and China (Table 2). Symptoms on these bast fiber crops include dark brown and often fusiform to ellipsoidal or irregularly shaped spots on leaves, petioles, and stems. New leaves and shoots are among the most susceptible to anthracnose infections [24,25,26,27,28,29,30].

Pathogens from the oomycete genus *Pythium* cause crown rot and root rot in both ramie and hemp crops as well as in marijuana plants grown in both field and hydroponic conditions [31,32]. Five species from this genus are pathogenic against ramie and hemp plants: *Pythium vexans*, *Pythium dissotocum*, *Pythium myriotylum*, *Pythium aphanidermatum*, and *Pythium ultimum*. Both crown and root rots are more common in cool conditions than in hot conditions, particularly in low lying or flood-prone areas where hemp is intensively grown. Findings from field tests indicate *P. aphanidermatum* could infect different tissues and organs of cannabis plants, resulting in rot, wilt, and eventual collapse of the whole host plant [31,32,33,34,35,36].

Fungi from the genus *Verticillium* are also persistent pathogens affecting the xylem vessels of susceptible plants. *Verticillium* fungi can survive for a long time in the soil [37]. At present, three *Verticillium* species are known to be associated with flax: *Verticillium dahliae*, *Verticillium tricorpus*, and *Verticillium longisporum*. Among these three, *V. dahliae* is the only confirmed pathogen of flax crop. This pathogen can cause flax wilt and lead to non-negligible yield losses and depreciated fibers, both of which are difficult to deal with after harvesting. *Verticillium* wilt caused by *V. dahliae* often triggers wilt and necrosis in the leaves, brown discoloration of epidermis, and vascular tissues in the main root and stem. *V. dahliae* can form microsclerotia that are resistant to a variety of stresses and be easily dispersed to other fields by hiding in host debris [36,37,38]. Two other *Verticillium* species, named *V. tricorpus* and *V. longisporum*, have been found in the flax culture soil, but their pathogenicity to flax remains to be determined [38].

Wilting and crown rot diseases, caused by fungi from the genus *Fusarium* and often accompanied by vascular and pith discoloration symptoms, are among the most devastating diseases in bast fiber crops. *Fusarium* pathogens of bast fiber crops are present mainly in the *F. oxysporum* species complex (FOSC) [39,40,41]. The reported diseases on bast fiber crops associated with FOSC include jute brown wilt, hemp wilt, and crown rot. Several other *Fusarium* species, such as *Fusarium solani*, *Fusarium brachygibbosum*, and *Fusarium udum f. sp. crotalariae* can also cause wilt and crown rot in hemp, jute, and sunn hemp. *Fusarium* spp. are common residents in agricultural soils and can live a saprophytic lifestyle. However, most *Fusarium* species can cause diseases in a diversity of plants, including bast fiber crops [39,40,41]. At present, the genetic basis for the broad host range of *Fusarium* species is largely unknown. However, their broad host ranges and ability to grow and survive in a diversity of environments makes it very challenging to prevent and control these pathogens.

In recent years, powdery mildew on hemp and sunn hemp, caused by fungal pathogens from the genus *Golovinomyces*, were observed on indoor or field-grown plants in multiple locations in North America. The species from this genus that cause diseases in bast fiber crops include *Golovinomyces cichoracearum*, the agent of sunn hemp powdery mildew, and *Golovinomyces spadiceus* and *Golovinomyces cichoracearum sensu lato*, which are responsible for powdery mildew on hemp. Similarly, *Podosphaera macularis* and *Leveillula taurica* can also cause hemp powdery mildew [42,43,44]. Hemp powdery mildew ranges in incidence from 20% to 35% on several varieties. The disease symptoms first appear as inconspicuous white patches on leaves and stems. As the disease progresses, colonies like mycelia, conidiophores, and conidia may expand and spread to flower bracts and buds, including those of other plants. The disease spreads readily to asymptomatic hosts [42,43,44,45].

Fungi from the genus *Alternaria* have a relatively limited host range among bast fiber crops. *Alternaria alternata* can cause leaf spot diseases in ramie and cannabis plants [46,47]. The disease symptoms often appear as small brown or circular spots in the leaves on cannabis plants, or as irregular and necrotic lesions on ramie leaves. The disease incidence ranges from 11.8%–30% in southern China. Productivity-wise, black leaf spot in ramie, caused by *A. alternata*, may reduce the yields of leaves and shoots by 20%–50% [46,47].

Aside from the major fungal genera mentioned above that have been identified as causal agents of significant diseases in bast fiber crops, other fungal pathogens, such as *Cercospora* cf. *flagellaris* [48]*, Exserohilum rostratum* [49]*, Macrophomina phaseolina* [50]*, Sclerotinia minor* [51], *Micropeltopsis cannabis sp.*, *Orbilia luteola*, *Curvularia cymbopogonis* [52]*, Podosphaera xanthii* [53], and *Lasiodiplodia theobromae* [54,55], can also cause a diversity of known or unknown diseases among bast fiber crops. The diseases include leaf spot, foliar blight, charcoal rot, sclerotinia crown rot in industrial hemp, powdery mildew on ramie, and black rot on kenaf, resulting in different degrees of damage to productivity and quality [48,49,50,51,52,53,54,55]. The details are shown in Table 2.

## 4. Development of Molecular Identification of Bast Fiber Fungal Pathogens

At present, most diagnosis of bast fiber diseases rely on disease symptoms and, when available, cultural characteristics of isolated fungal pathogens on artificial media. However, it is often difficult to identify the underlying pathogen based on those characters alone. For example, the disease symptoms of *Verticillium* wilt in hemp is very similar to *Fusarium* wilt and the pathogen species in both genera can invade a wide range of economical crops [37,38,39]. In addition, it is difficult to distinguish the species within most fungal genera based on morphological features alone. However, most of them are relatively easy to identify using molecular markers, as described below (Table 2; Table 3). 

**Figure 1:**
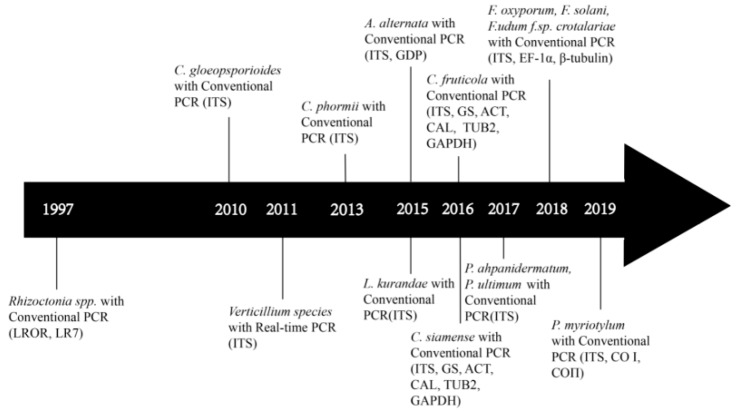
Development of molecular-based assays for the detection of fungal pathogens in bast fiber crops from 1997 until the present. For genus and species names, please see text and Table 2. Details of primers are shown in Table 3.

As early as 1997, a PCR-based method was used to help identify fungal pathogens of bast fiber crops. Specifically, McPartland et al. [52] amplified part of the 28S ribosomal RNA (rRNA) gene followed by *EcoR I/Hind III* digestion and electrophoresis to differentiate hemp fungal pathogens, and named two new species: *Micropeltopsis cannabis* sp. nov. and *Orbilia luteola* (Roum.) comb. nov. However, there were relatively few reports of fungal pathogens on bast fiber crops between 1998 and 2009, likely due to limited production of bast fiber crops and an emphasis on chemical fiber and other natural fibers. During this period, the acreage and production of bast fiber crops were low and there was limited research on these crops. Since 2009, with increasing production and research on bast fiber crops, there have been increasing reports on infectious diseases, including fungal diseases, on these crops [23]. This is especially true over the last five years when a large number of fungal pathogens were reported from bast fiber crops and many of these were identified based on molecular markers (Figure 1).

According to the National Center for Biotechnology Information (NCBI) PubMed, the most common literature on the molecular identification of fungal pathogens on bast fiber crops has been on hemp (including both industrial hemp and medicinal marijuana), accounting for ~45% of all published articles. This was then followed by flax and kenaf (at ~14% each), ramie (11%), and the rest being jute and sunn hemp. However, most of these reports were case reports. Below we present a summary review on this topic.

## 5. Target DNA Selection and Molecular Assays of Fungal Pathogens on Bast Fiber Crops

Over the last three decades, several types of DNA-based methods have been developed and widely used to detect plant fungal pathogens. The invention of PCR technology using a thermostable polymerase by Kary Mullis gave birth to PCR in the early 1980s [4]. The invention of PCR has led to a diversity of PCR-based methods for fungal pathogen detections based on variations in DNA sequences within and among species (Figure 1, Table 2). Among these methods, qPCR is probably the most common molecular technology and it can be used for quantitative measurement of RNA and DNA, targeting both single nucleotide polymorphisms (SNPs) and copy number variations. qPCR allows not only the detection of whether a specific pathogen(s) is present in the sample, but also the quantification of pathogen levels in host tissues [5,6]. To improve the efficiency of conventional PCR, other methods have been coupled with PCR for plant fungal pathogen detection. For example, PCR in combination with enzyme-linked immunosorbent assay (ELISA) has been successfully applied to detect fungi, viruses, and bacteria, with high specificity [56]. Similarly, the highly specific IC-PCR approach can increase the sensitivity by 250 folds compared to conventional PCR amplification [7,8]. For absolute quantification without the need for references and standard curves, dd-PCR is the method of choice—this method is based on the combined technology of water–oil emulsion droplet and PCR [9]. In field conditions without ready access to laboratory equipment, LAMP can provide fast identifications of samples. LAMP uses six primers that are highly specific to target sites in a specific gene [10]. It can be carried out at a constant temperature in a short reaction time (<30 min). It is sensitive and cost-effective, potentially making it an ideal method for field detection of plant pathogens [57].

As shown in Table 2, PCR-based methods have been used as the main approach for detecting fungal pathogens in bast fiber crops. This pattern is similar to the detections of fungal pathogens in other crops in general. A number of DNA fragments and genes have been explored as potential targets for PCR-based detections, including the ribosomal RNA gene cluster, conserved housekeeping genes, and genes involved in the production of secondary metabolites, including mycotoxins [58,59,60]. Table 3 summarizes the genes and their primers that have been used for the detection and diagnostics of fungal pathogens on bast fiber crops. We would like to note that the molecular analyses reported so far for identifying fungal pathogens on bast fiber crops have been primarily using pure fungal strains, not those from diseased plant tissues. There is a large gap in applying these molecular methods in field conditions as a point-of-care test.

**Figure 2:**
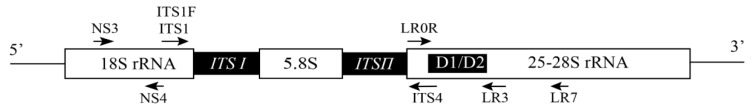
A schematic representation of the fungal ribosomal RNA gene cluster showing the locations of individual DNA fragments and the common primers used for PCR amplification. Adapted from White et al. (1990) and Adam et al. (2017).

Among the DNA fragments that have been used for fungal pathogen detection, the most frequently used is the ribosomal RNA gene cluster. This gene cluster is composed of up to hundreds of repeating units with each unit containing the genes encoding the small (18S) ribosomal RNA subunit, the internal transcribed spacer (ITS) regions 1 and 2 that are separated by the 5.8S rRNA subunit, and the large (28S) ribosomal RNA subunit, with the intergenic spacer (IGS) region separating the adjacent units (Figure 2). The entire ITS fragment (which comprises ITS1, 5.8S rRNA, and ITS2) is typically 500–750 bp long and flanked by the 18S and 28S rRNA genes [61,62,63]. The ITS regions are present in all known fungi and have both highly conserved flanking regions located in the 5.8S, 18S, and 28S rRNA genes as well as the variable regions (located in the ITS1 and ITS2 regions). The conserved flanking regions allowed the development of highly conserved probes or primers to amplify most, if not all, fungi, while the variable regions allowed the development of species-specific markers [64,65]. Together, these features have contributed to ITS being the consensus fungal DNA barcode for the mycological community [64,65]. Furthermore, the ITS sequences obtained from the direct amplification and sequencing of environmental DNA samples have contributed to our increased understanding of fungal diversity from a variety of environments, including those from diseased plants and animals [65,66].

Indeed, our literature analysis showed that over 80% of fungal pathogens infecting bast fiber crops were identified based on PCR-based assays targeting the ITS regions. For example, Wang et al. [29] first identified *C. gloeosporioides* and *C. higginsianum* as the agents of anthracnose disease on ramie plants in China based on ITS sequences [29]. Similarly, Serdani et al. [24,25] obtained ITS sequences and reported that *C. phormii* was the main agent causing anthracnose on New Zealand flax in the United States and Australia [24,25]. Other fungal pathogens infecting bast fiber crops identified based on ITS sequences include *G. cichoracearum*, *A. alternata*, *P. aphanidermatum*, *P. ultimum*, and *F. oxysporum* etc. [35,40,44,46]. Aside from species identification, variations in ITS sequences have also been used to reveal new species and understand the relationships among broad groups of fungi, including those causing diseases in bast fiber crops. For example, based on ITS sequence information, Kwon et al. [28] found a potential new species in the genus *Colletotrichum* causing kenaf anthracnose disease outbreaks in Korea during the summers of 2013 and 2014 [28]. This new species had an ITS sequence distinct from *Colletotrichum acutatum*, its closest related species.

However, in certain situations, ITS sequences alone are insufficient for species identification and for revealing the phylogenetic relationships among strains and species of fungi. Thus, it is common nowadays to combine ITS sequence information with those from other gene fragments. Several genes have been commonly used, including those coding for translation elongation factor 1-α (*TEF1-α* or *EF-1α*) [32], ß-tubulin (*TUB*) [42], calmodulin (*CAL*) [50], histone subunit 3 (*H3*) [48], glyceraldehydes-3-phosphate dehydrogenase (*GAPDH*) [47], and actin (*ACT*) [48]. By sequencing the ITS and 28S regions with primers PM5G/NLP2, Szarka et al. [43] made the first documented report of *G. spadiceus* causing powdery mildew on industrial hemp in the United States [43]. Yu et al. identified *A. alternata* as the agent of black leaf spot disease on ramie plants in China on the basis of morphology and DNA sequences at the *ITS* and *GAPDH* genes [47]. Similarly, based on the phylogenetic analysis of *ITS* and *EF-1α*, Zamir et al. [32] revealed that several *Fusarium* and *Pythium* species affecting cannabis plants shared 99%–100% sequence identities with isolates causing stem rot and wilt in other plants, consistent with the broad host range of many of these plant fungal pathogens.

Within the ribosomal RNA gene cluster, aside from the ITS regions, several other regions including 18S, 5.8S, 28S, and IGS regions, have also been used as PCR targets for the detection of fungal pathogens in bast fiber crops [61]. Each of these gene fragments contain relatively conserved regions that are valuable for species-specific primer designs and variable regions for studying related taxonomic groups [65]. In 1997, based on sequencing of a region of the 28S rRNA gene with primers LROR and LR7, McPartland et al. [52] proposed two new species *Micropeltopsis cannabis* sp. nov. and *Orbilia luteola* comb. nov., revised the list of five new fungal–*Cannabis* associations, and reported three known fungal pathogens impacting *Cannabis* plants for the first time at several new locations [52].

After *ITS*, the *EF-1α* gene is the second most common target gene for molecular diagnosis of bast fiber fungal pathogens (Table 2). The *EF-1α* gene is a single-copy nuclear protein-coding gene with highly conserved sequences among species. It is a secondary DNA barcode for many groups of fungi [65], often used in phylogenetic studies of divergent fungal groups. Although the database of *EF-1α* sequences is not as large as that for ITS sequences, *EF-1α* often contains more variable nucleotide sites than that of ITS and thus can be particularly useful for separating closely related organisms [65,66]. For example, the ITS sequences are often not informative for distinguishing closely related species in *Fusarium* while on the other hand, *EF-1α* sequences can [66]. Zamir et al. [32,34] compared *F. oxysporum* and *F. brachygibbosum* isolates from cannabis plants in northern California (USA) with all other *Fusarium formae speciales* and isolates previously recovered from British Columbia (Canada) using sequences at *EF-1α* and ITS regions [32,34]. They found that a diversity of fungal pathogens can cause hemp root and crown rot under field conditions. However, phylogenetic analysis of *EF-1α* and ITS sequences revealed that *Fusarium* species, such as *F. oxysporum* and *F. solani*, were the primary fungal pathogens impacting hydroponically grown cannabis plants and that these strains of *F. oxysporum* and *F. solani* shared 99%–100% sequence identity with those causing stem rot and wilt in other host plants. Similarly, based on *EF-1α* and *CAL* sequences, Casano et al. [50] identified that *Macrophomina phaseolina* was the agent of charcoal rot on hemp cultivated in southern Spain [Table 2].

As an important component of the cytoskeleton and spindle microtubules, the highly conserved ß-tubulin gene has been another marker gene for identifying plant fungal pathogens [67]. For example, in 2011, Debode et al. [39] developed a qPCR method to detect several species in *Verticillium* based on the ß-tubulin gene [39]. Their method allowed the differentiation of the species *V. tricorpus*, *V. dahliae*, and *V. longisporum* in one day. On the other hand, Wang et al. relied on concatenated sequences of *ß-tubulin* and *EF-1α* sequences to identify a new sub-species of *F. udum f. sp. Crotalariae* causing sunn hemp *Fusarium* wilt in Taiwan [42]. 

As mentioned above and reviewed elsewhere (e.g., [65]), several other genes have also been used as target DNA in molecular identification for fungal pathogens [68,69]. Those frequently utilized DNA fragments are in conserved genes such as *CAL*, *H3*, *GDP*, and *ACT*. However, these genes generally appeared in the form of multi-locus sequence typing (MLST) [70,71], and few are used individually in the diagnosis of fungal pathogens in bast fiber crops.

MLST is also known as multi-locus sequence analysis (MLSA) or multiple gene genealogical analysis (MGGA) [70,71]. As the name suggests, MLSA usually analyzes a combination of multiple (usually five to seven) genes (typically housekeeping genes) for each strain. As a result, polymorphisms from all gene fragments can be used together to allow for greater discrimination of strains and species than single gene sequences [71,72,73]. This approach has been used by Doyle et al. [48] in their analysis of fungal pathogens causing leaf spot diseases in industrial hemp fields in Kentucky in 2015. Specifically, they obtained sequences for parts of *CAL*, *H3*, *ACT*, and *EF1-α* genes and the complete ITS sequences for their strains and identified *Cercospora* cf. *flagellaris* as the causal agent of leaf spot disease in their fields [48]. Similarly, based on sequences at six loci (*ACT*, *TUB2*, *CAL*, *GS*, *GAPDH*, and *ITS*), Niu et al. revealed that *C. fructicola*, *C. siamense*, and *C. corchorumcapsularis sp. nov*. were associated with jute anthracnose in southeastern China [26,27].

## 6. Conclusions and Future Prospects

As shown above, fungal pathogens of bast fiber crops are very diverse in their taxonomic distributions, ecological niches, and host ranges. Most of these fungi can exist both as saprophytes and as pathogens. Morphologically, they can exist in different forms such as the sexual or asexual spore form and/or the hyphal form (for filamentous and dimorphic fungi). Physiologically, these fungi may be highly active, growing and dividing exponentially, or inactive, in dormant/resting state in natural environments. In addition, they may exist at very high or very low concentrations, or anywhere in between. Some of these fungi are easily culturable in the lab while others may be difficult to cultivate or even unculturable. Most microbial communities in nature, including those on diseased bast fiber crops, often contain a mixture of bacterial and fungal species. As a result, it can be extremely challenging to isolate certain fungal pathogens and identify them based on their cultural and other characteristics. Thus, having a molecular-based method can significantly enhance the detection and diagnosis of pathogens directly from the environment, including diseased plants.

At present, even though progress has been made in the development of molecular diagnosis tools, most diagnoses of bast fiber diseases still rely on disease symptoms and, when available, cultural characteristics on artificial media. However, these features are often not species-specific and often take a long time to obtain. One big advantage of molecular methods over traditional methods is that the molecular methods can be directly applied to plant and soil samples to obtain early detection of potentially devastating and persistent fungal pathogens, even when physiological symptoms are not visible or in a latent phase on crops. Furthermore, molecular markers can contribute to revealing variations among strains of fungal pathogen populations, including virulence and toxin-producing genes. Indeed, the potential advantages of molecular-based technologies for fungal pathogen detection and identification are enormous.

Over the past few years, effective amplification platforms, probe development, and various quantitative PCR technologies have revolutionized research on fungal pathogen detection. From basic research to point-of-care diagnosis, the latest assays and technologies have laid a solid foundation for developing effective fungal detection systems, including those for bast fiber crop-specific fungal diseases. At present, the detection and diagnosis of fungal pathogens in bast fiber crops lag far behind those for several other groups of fungi. Although many types of molecular assays have been developed in recent years, most still rely on pure fungal cultures and require sophisticated equipment. At present, the dominant molecular methods for diagnosing fungal pathogens on bast fiber crops are conventional PCR followed by DNA sequencing. Indeed, there were only a few instances where the more sensitive and species-specific real-time PCR methods were developed for direct identification of fungal pathogens. Future research should aim to develop cost-effective methods that can work directly on diseased plant tissues in field conditions. Indeed, there is great potential for the development of technologies targeting fungal pathogens of bast fiber crops in field conditions. The potential technologies include IC-PCR, PCR-ELISA, dd-PCR, multiplex PCR, LAMP, and DNA microarray [14]. These approaches can increase both the sensitivity and specificity of molecular detection systems. With increasing genomic information from these fungal pathogens, unique markers for each species (or even sub-species) should be easily identifiable and be developed for effective identification of fungal pathogen species and genotypes infecting bast fiber crops.

## Figures and Tables

**Table 1:** Major types of bast fiber crops and their distributions around the world [20,21,22].

Crop	Main Distribution	Main Characters of Growth Habitat	Main Applications	Main Fungal Diseases
Flax (*Linum usitatissimum* Linnaeus)	France, Russia, Netherlands, Belarus, Belgium, Canada, Kazakhstan, China, India	Well-drained loam and cool, moist, temperate climates	Linen, flax yarn, flax seed, linseed oil	flax wilt, flax blight, flax anthracnose
Hemp (*Cannabis sativa* Linnaeus)	China, Canada, USA, Europe, East Asia, Nepal	Grows at 16–27 °C, sufficient rain at the first six weeks of growth, short day length.	Textiles, hempseed oil, prescription drugs	hemp powdery mildew, hemp leaf spot disease, hemp blight, hemp root and crown rot wilt, hemp charcoal rot
Jute (*Corchorus capsularis* Linnaeus)	India, Bangladesh, Burma, China	Tropical lowland areas, humidity of 60% to 90%, rain-fed crop	Textiles, medicine	jute anthracnose, jute brown wilt, jute leaf spot
Kenaf (*Hibiscus cannabinus* Linnaeus)	India, Bangladesh, China, Malaysia, Thailand	Sandy loam and warm, humid subtropical, or tropical climates, few heavy rains or strong winds, at least 12 h light each day	Textiles	kenaf anthracnose, kenaf lack rot, kenaf sooty mold
Ramie (*Boehmeria nivea* Linnaeus) Gaudich	China, Brazil, Philippines, India, Vietnam, Laos, Cambodia	Sandy soil and warm, wet climates, rainfall averaging at least 75 to 130 mm per month	Textiles, soil and water conservation, medicine	ramie anthracnose, ramie powdery mildew, ramie black leaf spot, ramie blight
Sunn Hemp (*Crotalaria juncea* Linnaeus)	India, USA, China	Wide variety of soil condition, altitude from 100 to 1000 m, temperatures above 28 °C, photoperiod-sensitive	Cover crop or green manure, forage producer	sunn hemp fusarium wilt, sunn hemp root rot, sunn hemp powdery mildew

**Table 2:** List of fungal pathogens on bast fiber crops identified using molecular method.

*Pathogen*		Disease	Method	Marker	Host Plant	Geographic Region(s)	Reference
***Alternaria***							
*A. alternata*		Hemp leaf spot	Conventional PCR	ITS	*Cannabis sativa*	Shanxi, China	[46]
*A. alternata*		Ramie black leaf spot	Conventional PCR	ITS, GAPDH	*Boehmeria nivea*	Hunan, Hubei, China	[47]
***Cercospora***							
*Cercospora cf. flagellaris*		Hemp leaf spot disease	Not mentioned	ITS, EF-1α, CAL, H3, actin	*Cannabis sativa*	Kentucky, USA	[48]
***Colletotrichum***							
*C. corchorum capsularis*		Jute anthracnose	Conventional PCR	ACT, TUB2, CAL, GAPDH, GS, and ITS	*Corchorus capsularis L*.	Zhejiang, Fujian, Guangxi, and Henan, China	[27]
*C. fructicola*		Jute anthracnose	Conventional PCR	ACT, TUB2, CAL, GAPDH, GS, and ITS	*Corchorus capsularis L*.	Zhejiang, Fujian, Guangxi, and Henan, China	[26]
*C. fructicola*		Jute anthracnose	Conventional PCR	ACT, TUB2, CAL, GAPDH, GS, and ITS	*Corchorus capsularis L*.	Zhejiang, Fujian, Guangxi, and Henan, China	[27]
*C. gloeosporioides*		Ramie anthracnose	Conventional PCR	ITS	*Boehmeria nivea*	HuBei, HuNan, JiangXi, and SiChuan, China	[30]
*C. higginsianum*		Ramie anthracnose	Conventional PCR	ITS	*Boehmeria nivea*	HuBei, China	[29]
*C. phormii*		New Zealand flax anthracnose	Conventional PCR	ITS	*Phormium tenax*	California, USA	[24]
*C. phormii*		New Zealand flax anthracnose	Conventional PCR	ITS	*Phormium tenax*	Perth, Australia	[25]
*C. siamense*		Jute anthracnose	Conventional PCR	ACT, TUB2, CAL, GAPDH, GS, and ITS	*Corchorus capsularis L*.	Zhejiang, Fujian, Guangxi, and Henan, China	[26]
*Colletotrichum sp*.		Kenaf anthracnose	Conventional PCR	ITS	*Corchorus olitorius*	South Korea	[28]
***Curvularia***							
*C. cymbopogonis*		Hemp leaf spot	Conventional PCR	25S	*Cannabis sativa*	USA	[52]
***Exserohilum***							
*E. rostratum*		Hemp floral blight	Not mentioned	ITS, RPB2	*Cannabis sativa*	North Carolina, USA	[49]
***Fusarium***							
*F. oxysporum*		Hemp roots and crown rot	Conventional PCR	ITS, EF-1α	*Cannabis sativa*	Canada	[32]
*F. oxysporum*		Jute brown wilt	Conventional PCR	ITS	*Corchorus olitorius*	Dhaka, Manikgonj, Kishorgonj, Rangpur, and Monirampur, Bangladesh	[40]
*F. oxysporum*		Hemp wilt	Conventional PCR	ITS, EF-1α	*Cannabis sativa*	California, USA	[34]
*F. solani*		Hemp crown root	Conventional PCR	ITS, EF-1α	*Cannabis sativa*	Canada	[32]
*F. solani*		Hemp wilt	Conventional PCR	ITS, EF-1α	*Cannabis sativa*	California, USA	[34]
*F. solani*		Sunn hemp root rot and wilt	Conventional PCR	ITS, EF-1α	*Crotalaria juncea*	Ceará, Brazil	[41]
*F. brachygibbosum*		Hemp wilt	Conventional PCR	ITS, EF-1α	*Cannabis sativa*	California, USA	[34]
*F. udum f. sp. crotalariae*		Sunn hemp fusarium wilt	Conventional PCR	EF-1α, β-tubulin	*Crotalaria juncea*	Tainan, China	[42]
***Glomus***							
*G. mosseae*		Hemp root rot	Conventional PCR	25S	*Cannabis sativa*	USA	[52]
*Golovinomyces*							
*G. spadiceus*		Hemp powdery mildew	Not mentioned	ITS, 28S	*Cannabis sativa*	Kentucky, USA	[43]
*G. cichoracearum sensu lato*		Hemp powdery mildew	Conventional PCR	ITS	*Cannabis sativa*	Atlantic Canada and British Columbia.	[44]
*G. cichoracearum*	*Sunn hemp powdery mildew*		Not mentioned	ITS	*Crotalaria juncea*	Florida, USA	[45]
***Lasiodiplodia***							
*L. theobromae*		Kenaf black rot	Conventional PCR	ITS	*Corchorus olitorius*	Kangar Perlis, Malaysia	[54]
*Leptoxyphium*							
*L. kurandae*		Kenaf sooty mould	Conventional PCR	ITS	*Corchorus olitorius*	Iksan, Korea	[55]
***Macrophomina***							
*Macrophomina phaseolina*		Hemp charcoal rot	Conventional PCR	EF-1α, CAL	*Cannabis sativa*	Southern Spain	[50]
***Micropeltopsis***							
*Micropeltopsis cannabis*		Unknown	Conventional PCR	25S	*Cannabis sativa*	USA	[52]
***Orbilia***							
*Orbilia luteola*		Unknown	Conventional PCR	25S	*Cannabis sativa*	USA	[52]
***Pestalotiopsis***							
*Pestalotiopsissp*.		Hemp spot blight	Conventional PCR	25S	*Cannabis sativa*	USA	[52]
***Podosphaera***							
*P. xanthii*		Ramie powdery mildew	Conventional PCR	ITS	*Boehmeria nivea*	Naju, Korea	[53]
***Pythium***							
*P. dissotocum*		Browning and a reduction in root mass, stunting	Conventional PCR	ITS, EF-1α	*Cannabis sativa*	Canada	[32]
*P. myriotylum*		Browning and a reduction in root mass, stunting	Conventional PCR	ITS, EF-1α	*Cannabis sativa*	Canada	[32]
*P. myriotylum*		Hemp root rot and Wilt	Conventional PCR	ITS, COI, COII	*Cannabis sativa*	Connecticut, USA	[33]
*P. aphanidermatum*		Hemp root rot and crown wilt	Conventional PCR	ITS	*Cannabis sativa*	California, USA	[34]
*P. aphanidermatum*		Hemp crown and root Rot	Conventional PCR	ITS	*Cannabis sativa*	Indiana, USA	[35]
*P. ultimum*		Hemp crown and root Rot	Conventional PCR	ITS	*Cannabis sativa*	Indiana, USA	[36]
***Rhizoctonia***							
*Binucleate R. spp*.		Hemp wilt	Conventional PCR	25S	*Cannabis sativa*	USA	[52]
***Sclerotinia***							
*Sclerotinia minor*		Hemp crown rot	Conventional PCR	ITS	*Cannabis sativa*	San Benito County, Canada	[51]
***Sphaerotheca***							
*S. macularis*		Hemp powdery mildew	Conventional PCR	25S	*Cannabis sativa*	USA	[52]
***Verticillium***							
*V. dahliae*		flax wilt	Conventional PCR	ITS	*Linum usitatissimum*	La Haye Aubrée, France	[37]
*V. dahliae*		flax wilt	qPCR	ITS	*Linum usitatissimum*	Normandy, France	[38]
*V. dahliae*		flax wilt	qPCR	ß-tubulin	*Linum usitatissimum*	Germany	[39]
*V. tricorpus*		flax wilt	qPCR	ITS	*Linum usitatissimum*	Germany	[39]
*V. longisporum*		flax wilt	qPCR	ß-tubuIin	*Linum usitatissimum*	Germany	[39]

qPCR: quantitative PCR, ITS: internal transcribed spacer, GAPDH: glyceraldehydes-3-phosphate dehydrogenase, GS: glutamate synthetase, EF-1α: translation elongation factor 1-α, CAL: calmodulin, H3: histone subunit 3, ACT: actin, TUB2: ß-tubulin, RPB2: RNA polymerase subunit B2, COI: cytochrome oxidase subunit I, COII: cytochrome oxidase subunit II.

**Table 3:** Genes and PCR primers used for their amplification in fungal pathogens infecting bast fiber crops.

Target DNA	Primer Name and Sequence (5′-3′)		Size of PCR Product (bp)	Reference
*18S*	NS3	GCAAGTCTGGTGCCAGCAGCC	Not mentioned	[31]
	NS4	CTTCCGTCAATTCCTTTAAG		
*28S*	LR0R	GCAAGTCTGGTGCCAGCAGCC	Not mentioned	[31]
	LR3	GCAAGTCTGGTGCCAGCAGCC		
*25S*	LROR	ACCCGCTGAACTTAAGC	1431	[52]
	LR7	TACTACCACCAAGATCT		
*ACT*	ACT-512F	ATGTGCAAGGCCGGTTTCGC	300	[48]
	ACT-783R	TACGAGTCCTTCTGGCCCAT		
*ß-tubulin*	Vd-btub-1F	GCGACCTTAACCACCTCGTT	Not mentioned	[38]
	Vd-btub-1R	CGCGGCTGGTCAGAGGA		
	VertBt-F	AACAACAGTCCGATGGATAATTC	Not mentioned	[38]
	VertBt-R	GTACCGGGCTCGAGATCG		
	VITubF2	GCAAAACCCTACCGGGTTATG	143	[39]
	VITubRl	AGATATCCATCGGACTGTTCGTA		
	VdTubF2	GGCCAGTGCGTAAGTTATTCT	82	[39]
	VdTubR4	ATCTGGTTACCCTGTTCATCC		
	Bt2a	GGTAACCAAATCGGTGCTGCTTTC	Not mentioned	[26]
	Bt2b	ACCCTCAGTGTAGTGACCCTTGGC		
*CAL*	CL1	GARTWCAAGGAGGCCTTCTC	Not mentioned	[26]
	CL2	TTTTTGCATCATGAGTTGGAC		
	CAL-228F	GAGTTCAAGGAGGCCTTCTCCC	Not mentioned	[50]
	CAL-737R	CATCTTTCTGGCCATCATGG		
*EF-1α*	EF-1	ATGGGTAAGGAGGACAAGAC	700	[34]
	EF-2	GGAGGTACCAGTGATCATGTT		
	EF1-728F	CATCGAGAAGTTCGAGAAGG	Not mentioned	[50]
	EF2	GGAGGTACCAGTGATCATGTT		
	EF1-728F	CATCGAGAAGTTCGAGAAGG	350	[48]
	EF1-983R	TACTTGAAGGAACCCTTACC		
*Endochitinase*	Vd-endoch-1F	CTCGGAGGTGCCATGTACTG	Not mentioned	[38]
	Vd-endoch-1R	ACTGCCTGGCCCAGGTTC		
*GAPDH*	Vd-G3PD-2F	CACGGCGTCTTCAAGGGT	Not mentioned	[38]
	Vd-G3PD-1R	CAGTGGACTCGACGACGTAC		
	GDF1	GCCGTCAACGACCCCTTCATTGA	Not mentioned	[26]
	GDR1	GGGTGGAGTCGTACTTGAGCATGT		
	gpd-1	CAACGGCTTCGGTCGCATTG	Not mentioned	[47]
	gpd-2	GCCAAGCAGTTGGTTGTGC		
*GS*	GSF1	ATGGCCGAGTACATCTGG	Not mentioned	[26]
	GSR1	GAACCGTCGAAGTTCCAC		
*ITS*	ITS1	TCCGTAGGTGAACCTGCGG	334-738	[24,30,35,36,37,38]
	ITS4	TCCTCCGCTTATTGATATGC		
	Vd-ITS1-45-F	CCGGTCCATCAGTCTCTCTG	334	[37]
	Vd-ITS2-379-R	ACTCCGATGCGAGCTGTAAC		
	ITS1-F	CTTGGTCATTTAGAGGAAGTAA	700	[34]
	ITS4	TCCTCCGCTTATTGATATGC		
	VtF4	CCGGTGTTGGGGATCTACT	123	[39]
	VtR2	GTAGGGGGTTTAGAGGCTG		
	ITS 4	TCCTCCGCTTATTGATATGC	Not mentioned	[26]
	ITS 5	GGAAGTAAAAGTCGTAACAAGG		
*RPB2*	bRPB2-6F	TGGGGYATGGTNTGYCCYGC	Not mentioned	[49]
	bRPB2-7R	GAYTGRTTRTGRTCRGGGAAVGG		
